# The biomechanical effect of acupuncture for poststroke cavovarus foot: study protocol for a randomized controlled pilot trial

**DOI:** 10.1186/s13063-016-1264-x

**Published:** 2016-03-18

**Authors:** Yong Zhang, Hongwei Liu, Caihong Fu, Yanzhe Ning, Jiajia Zhang, Li Zhou, Zongheng Li, Peng Bai

**Affiliations:** Department of Rehabilitation, Dongzhimen Hospital, the First Affiliated Hospital of Beijing University of Chinese Medicine, No. 5, Haiyuncang, Dongcheng District Beijing, 100700 China; Department of Neurology, Dongzhimen Hospital, the First Affiliated Hospital of Beijing University of Chinese Medicine, No. 5, Haiyuncang, Dongcheng District Beijing, 100700 China; Department of Acupuncture, Dongzhimen Hospital, the First Affiliated Hospital of Beijing University of Chinese Medicine, No. 5, Haiyuncang, Dongcheng District Beijing, 100700 China

**Keywords:** Acupuncture, Biomechanical effect, Poststroke cavovarus foot, Study protocol

## Abstract

**Background:**

Poststroke cavovarus foot greatly affects patients’ activities of daily life and raises the risks of falls and consequent fractures. Acupuncture appears to be safe and effective in promoting motor functions and enhancing the activities of daily life among patients with poststroke cavovarus foot. The current study aims to study the biomechanical effect of acupuncture for poststroke cavovarus foot with objective outcome measurements.

**Methods/design:**

This is an assessor and analyst-blinded, randomized, controlled pilot study. A total of 60 eligible patients with poststroke cavovarus foot will be allocated by a 1:1 ratio into an acupuncture treatment group and a control group. Patients in the control group will receive conventional rehabilitation therapies, whereas a combination of acupuncture and conventional rehabilitation therapies will be applied in the acupuncture group. The primary outcome measures are three objective biomechanical parameters from the RSSCAN gait system: varus angle, dynamic plantar pressure distribution, and static plantar contact area. Scores of the Berg Balance Scale, the Fugl-Meyer Assessment, and the Stroke-Specific Quality of Life Scale, as well as other biomechanical parameters such as the step length and width, step time phase, and weight shifting phase will be selected as secondary outcome measurements. All assessments will be conducted at baseline, 4 weeks after the treatment course, and after a follow-up period of 3 months.

**Discussion:**

Results of the current study will provide detailed interpretations of the biomechanical effect of acupuncture for stroke rehabilitation and foundations for future larger clinical studies.

**Trial registration:**

Chinese Clinical Trial Registry: ChiCTR-IPC-15006889 (8 August 2015).

## Background

Stroke has become the major cause of disability and the second-most common cause of death worldwide [1]. An epidemiologic study published in 2007 indicated that China had more than 7 million stroke survivors, approximately 70 % of whom experienced functional disabilities [2]. Functional disabilities greatly influence the patients’ quality of life and leave a huge public health burden [3]. Poststroke cavovarus foot is one of the most frequent deformities among stroke survivors [4]. Studies showed that the incidence of poststroke cavovarus foot ranges from 17 % to 38 %, with 4 % to 9 % of the survivors suffering from disabling functions [5]. Poststroke cavovarus foot can generally reduce one's ability to walk, sit, stand, and undertake the activities of daily life and can also raise the risks of falls and consequent fractures [6].

A variety of rehabilitation techniques have been recommended for the management of poststroke cavovarus foot. Conventional therapies included positioning, splinting and casting, biofeedback, and electrical stimulation [4]. Botulinum toxin A, an approved pharmacological agent to treat poststroke spasticity, has also been increasingly used to treat poststroke cavovarus foot [7, 8]. However, in a recent study, Botulinum toxin A showed no better effect in improving limb functions when compared with conventional rehabilitation therapies in a majority of poststroke limb spasticity patients [9]. This reality drives us to search for additional effective modalities of treatment for poststroke limb spasticity and cavovarus foot.

As one of the most famous therapeutic modalities in Chinese medicine, acupuncture is becoming an important modality of alternative and complementary therapeutic intervention worldwide. Several clinical studies in China have been conducted to investigate the effect of acupuncture for poststroke cavovarus foot [10–13]. These previous studies indicated that, combined with conventional rehabilitation therapies, acupuncture is effective in promoting motor functions and enhancing the activities of daily life among patients with poststroke cavovarus foot [14]. However, the results of previous studies were limited to several shortcomings, such as failing to provide optimized acupuncture protocol and applying only clinical scales without objective outcome measurements.

In the current paper, we will provide detailed information of the rationale, design, and analytic methods of a pilot randomized controlled trial to investigate the biomechanical effect of acupuncture for poststroke cavovarus foot. We will try to study the effect of acupuncture for poststroke cavovarus foot with objective outcome measurements from the RSSCAN gait system.

## Methods/design

### Ethics

The protocol has been registered with the Chinese Clinical Trial Registry: ChiCTR-IPC-15006889. The study protocol has been approved by the Research Ethical Committee of Dongzhimen Hospital, the first affiliated teaching hospital of Beijing University of Chinese Medicine (No. ECPJ-BDY-2015-25). The Research Ethical Committee will also be in charge of supervising all procedures of our study, including patient recruiting, randomization, study conduct, data storage, and so on. In case of any changes to the study protocol, we will submit written application to the Research Ethical Committee. They will decide whether it is necessary or not to change the protocol.

### Study design

**Fig. 1:**
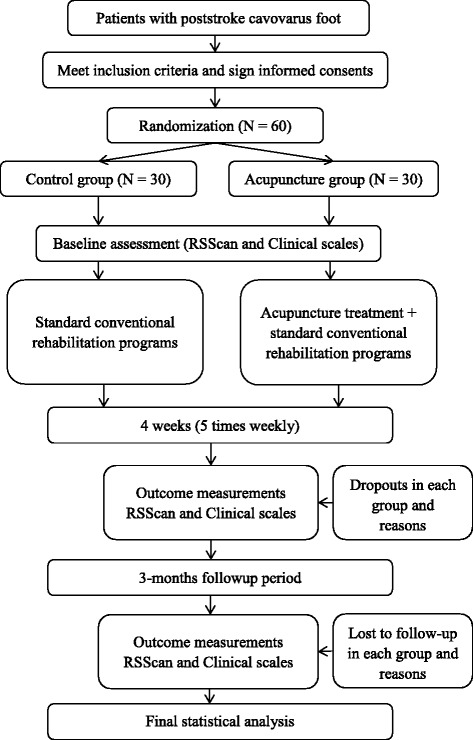
Flowchart of the study design

This is an assessor and analyst-blinded, randomized, controlled pilot study. The study will be conducted from 1 September 2015 to 31 December 2016 in Dongzhimen Hospital. Patients with poststroke cavovarus foot meeting the inclusion criteria will be allocated by a 1:1 ratio into an acupuncture treatment group or a control group. Patients in the control group will receive conventional rehabilitation therapies, whereas a combination of acupuncture and conventional rehabilitation therapies will be applied in the acupuncture group. Three objective biomechanical parameters, the varus angle, dynamic plantar pressure distribution, and static plantar contact area, from the RSSCAN gait system will be assessed as primary outcome measures. Other biomechanical parameters such as the step length and width, step time phase, and weight shifting phase, as well as scores of the Berg Balance Scale (BBS), the Fugl-Meyer Assessment (FMA), and the Stroke-Specific Quality of Life Scale (SSQOL) will be selected as secondary outcome measurements. All assessments will be conducted at baseline, 4 weeks after the treatment course, and after a follow-up period of 3 months. Figure 1 summarizes the study design.

### Inclusion criteria

Participants meeting the following inclusion criteria will be included: (1) ischemic stroke that have been confirmed by computed tomography (CT) or magnetic resonance imaging (MRI); (2) 40 to 75 years old; (3) first episode of stroke or with a history of stroke but with no serious deformity and modified Ranking Scale (mRS) grade ≤ 2; (4) 2 to 20 weeks after the onset of the current stroke; (5) with unilateral lower limb cavovarus foot and can walk at least 6 meters [15]; (6) blood pressure lower than 160/100 mmHg; (7) sufficient cognition to follow commands and Mini-Mental State Examination (MMSE) score > 24; and (8) voluntary participation and informed consent signed.

### Exclusion criteria

Participants with any of the following exclusion criteria will be excluded: (1) received surgery or thrombolytic therapy; (2) vital signs are not stable or show worsened conditions; (3) osteoarticular diseases of lower limbs, the foot, or the lumbar that would affect gait posture; (4) severe primary diseases of the cardiovascular system, hematopoietic system, kidney, or liver; (5) a pregnant or lactating woman; or (6) a participant in another clinical trial.

### Informed consent

Prior to the study, the general study process and the responsibilities of the participants and researchers will be explained to potential participants. Participants will be informed that their entry into the trial is entirely voluntary and that they could withdraw at any time. In the event of their withdrawal, data collected on the participant will not be erased and will be used in the final analyses. Written informed consent should be obtained from each participant before he or she undergoes any interventions related to the study.

### Randomization and allocation concealment

All participants will be assigned in a 1:1 ratio according to a computer-generated randomization list. Assignments will be sealed in opaque envelopes and will be opened by the study staff following informed consent procedures and baseline testing. The participants and researchers will know the allocation but the outcome assessors and data analysts will not.

### Interventions

#### Control group

Patients who are assigned to the control group will receive conventional rehabilitation programs for poststroke cavovarus foot, which consist of individualized amounts of various therapy options provided by occupational and physical therapists. Therapies are based on neurodevelopmental techniques and will include stretching, positioning, passive and active assisted lower limb activities, postural balance training, trunk control training, adaptive or compensatory strategies, and task-oriented practice. The rehabilitation programs will be carried out five times a week (that is, Monday to Friday) for 4 weeks, and every time the rehabilitation course will last approximately for 1 hour. All rehabilitation programs will be done by two qualified therapists.

#### Acupuncture group

In order to study the additional effects of acupuncture to conventional rehabilitation programs, patients allocated to the acupuncture group will receive acupuncture treatment in addition to control therapies.

The acupuncture treatment program is specially established for patients with poststroke cavovarus foot by an expert panel comprising a qualified senior acupuncturist from the department of acupuncture, an experienced therapist from the department of rehabilitation and a senior doctor from the department of Neurology.

The following acupoints from the stroke-affected side will be selected for needling: Qiuxu (GB40), Zhaohai (KI6), Kunlun (BL60), Taixi (KI3), Xuanzhong (GB39), Sanyinjiao (SP6); Yanglingquan (GB34); Yinlingquan (SP9). All acupoints are located according to the WHO standard acupuncture point locations in the Western Pacific Region. Disposable stainless steel acupuncture needles (0.25 × 40, Ande Co., Guizhou, China) will be manually inserted in an appropriate angle to a depth of 1.0-2.5 cm. Each acupuncture needle will be twisted until the patient felt a de-qi sensation and will be retained for 30 minutes. Acupuncture treatment will be performed by an independent certified practitioner with 8 years of clinical experience. The acupuncture treatment will take place 5 times a week (form Monday to Friday) and will last for 30 minutes every time.

#### Follow up

After the 4-week treatment observation, all patients will enter an additional 3-month follow-up period. Due to the specificity of stroke recovery, patients from both groups will be encouraged to attend community-based rehabilitation courses in the follow-up period. However, acupuncture treatment is not permitted for both groups. During the 3-month follow-up period, patients will be asked to fill out forms to record their rehabilitation attendance. All forms will be returned to the researchers for monitoring at the end of the trial.

#### Outcome measures

Data collection will be performed by a trained, certified assessor who is blind to patients’ assignment at baseline, after the intervention (4 weeks) and at the end of follow-up (3 months).

#### Basic characteristic variables

Demographic information including gender, age, ethnicity, time from the attack of stroke, use of medication and other detailed information will be collected at baseline to describe compare conditions and characteristics of two groups. Vital signs such as the resting blood pressure, pulse, respiration rate, and body temperature will be measured every day by nurses.

#### Primary outcomes

To the best of our knowledge, most previous studies applied only clinical scales to measure the outcomes of stroke rehabilitation. Few of them have introduced objective biomechanical outcome measurements. In the current study, we will try to provide several objective parameters by using the RSScan gait system (2 m × 0.4 m, 16,384 sensors, 480 Hz, RSScan International, Olen, Belgium). The system consists of a walking plate with sensor array, a data collector, and a computer. It is sensitive in detecting parameters of plantar pressure data and time phase when subjects are standing or walking on the sensor array plate. The RSScan system was first designed to test characteristics of athletes’ movements [16]. Recently, it has also been introduced into clinical studies to provide quantitative assessments [17, 18]. In the current study, we will collect data of varus angle, dynamic plantar pressure distribution, and static plantar contact area as primary outcome measures to evaluate the functions of lower extremity. Every time before formal data testing, patients will be asked to get familiar with the walking plate. All tests will be done by the same RSScan system operator.

#### Secondary outcomes

The BBS is an effective and appropriate assessment of balance in measuring outcomes of various stroke rehabilitation interventions [19]. It will be used as a secondary outcome measurement to assess the static and dynamic balance abilities of stroke patients in our study. The FMA will be applied as another secondary outcome measurement to assess the recovery of motor impairment. It includes an assessment of the upper extremity and lower extremity. In the current study, we will focus on the measurement of the lower extremity, which includes two subsections: the leg and coordination with a total score of 34. The SSQOL, a patient-reported assessment, will also be applied to assess the quality of life in both groups as secondary outcomes [20].

Some other objective parameters such as the step length and width, frequency of steps, speed of weight transition, and distribution of impulse from the RSScan system will also be listed as secondary outcome measurements.

#### Adverse Events

Any adverse events during the intervention period will be reported, and the causality with acupuncture therapy will be analyzed. All adverse events will be reported to the primary investigator and ethics committee to decide if the participant needs to withdraw from the trial. In case of stroke recurrence or other worsening conditions, the patients will be discontinued from the study and will be sent for further treatment.

#### Sample size

The current study is designed as a pilot study to investigate the biomechanical effects of acupuncture for poststroke cavovarus foot. A total of 60 patients, 30 in each group, will be recruited during the entire study. The results will provide evidence for the feasibility and sample size calculation of large-scale randomized controlled trials.

#### Statistical analysis

Statisticians who are independent from the research team will be responsible for the statistical analysis. The SPSS 12.0 software for Windows (Chicago, IL, USA) will be used. Categorical variables will be presented with frequencies or percentages and continuous variables will be presented as mean and standard deviation. The main analysis will compare the efficacy of acupuncture treatment group with the control group, including primary and secondary outcomes. Changes in all outcome measurements before and after the treatment and between groups will be analyzed. Demographic and clinical characteristics of two groups will be compared on admission using unpaired two-sample t-tests (continuous data) and Chi-square analysis (categorical data). Nonparametric methods will be used when assumptions of normality are violated. We will also conduct an intention-to-treat analysis if participants are lost to follow-up. The statistical significance threshold will be set at 0.05 (two-sided), with 95 % confidence intervals.

## Discussion

Acupuncture is a frequently used therapy for stroke rehabilitation in China, but the evidence of effect from previous studies seemed to be inconclusive [21]. Some systematic reviews have been done to study the effect of acupuncture for stroke rehabilitation [22–25]. These reviews have drawn consistent conclusions that acupuncture appeared to be safe and effective for stroke rehabilitation, but the benefits required further confirmation with larger, more transparent and well-conducted randomized clinical trials.

The complexity of stroke symptoms might be one of the possible reasons for previous studies and reviews concluding inconclusive results. A series of symptoms such as aphasia, apraxia, shoulder pain, cavovarus foot, limb numbness, and body imbalance will appear after the onset of stroke. It is difficult for acupuncture treatment to cover all symptoms of stroke rehabilitation. Thus, it would be reasonable to focus on certain aspects when investigating the effect of acupuncture for stroke rehabilitation. Previous studies and reviews indicated that acupuncture, when combined with conventional rehabilitation therapies, is effective in treating poststroke shoulder pain [26] and aphasia [27, 28], and improving poststroke imbalance [29] and quality of life [30]. In the current study, we will focus on the effect of acupuncture for cavovarus foot, which is one of the main poststroke symptoms risking patients’ daily life. We hope to reveal the real effect of acupuncture by focusing on one certain symptom rather than the whole stage of stroke rehabilitation.

Lack of objective quantitative assessments might be another reason affecting the quality of previous results. In the current study, we intend to study the effect of acupuncture for poststroke cavovarus foot with objective biomechanical outcome measurements from the RSScan gait system. The RSScan gait system was originally designed to provide objective biomechanical parameters for the adjustment of athletes’ movements to maximize their performance [16]. Recently, it has also been used in clinical studies of diabetes [17], multiple sclerosis [18], and knee osteoarthritis [31] to provide quantitative assessments and achieved satisfied results. Our group has tested the feasibility of applying RSScan gait system in the evaluation of stroke rehabilitation [32]. Thus, we believe that objective biomechanical results from the current study will provide more detailed interpretations of the effect of acupuncture for stroke rehabilitation, especially for poststroke cavovarus foot.

We present the protocol of a pilot randomized controlled trial aims at evaluating the biomechanical effect of acupuncture for poststroke cavovarus foot. Results of the current study will provide detailed interpretations of the biomechanical effect of acupuncture for stroke rehabilitation and foundations for future larger clinical studies.

### Trial status

This trial started on 1 September 2015 and is currently in the recruitment phase.

## Abbreviations

BBS: Berg balance scale
FMA: Fugl-Meyer Assessment
MMSE: Mini-Mental State Examination score
SS-QOL: Stroke-Specific Quality of Life Scale
